# Direct Detection of Akhiezer Damping in a Silicon MEMS Resonator

**DOI:** 10.1038/s41598-019-38847-6

**Published:** 2019-02-19

**Authors:** Janna Rodriguez, Saurabh A. Chandorkar, Christopher A. Watson, Grant M. Glaze, C. H. Ahn, Eldwin J. Ng, Yushi Yang, Thomas W. Kenny

**Affiliations:** 1 Department of Mechanical Engineering, Stanford University, Stanford, California 95304 USA; 2 Stanford Institute for Materials and Energy Sciences, SLAC National Accelerator Laboratory, Menlo Park, California, 94025 USA

## Abstract

Silicon Microelectromechanical Systems (MEMS) resonators have broad commercial applications for timing and inertial sensing. However, the performance of MEMS resonators is constrained by dissipation mechanisms, some of which are easily detected and well-understood, but some of which have never been directly observed. In this work, we present measurements of the quality factor, *Q*, for a family of single crystal silicon Lamé-mode resonators as a function of temperature, from 80–300 *K*. By comparing these *Q* measurements on resonators with variations in design, dimensions, and anchors, we have been able to show that gas damping, thermoelastic dissipation, and anchor damping are not significant dissipation mechanisms for these resonators. The measured *f* · *Q* product for these devices approaches 2 × 10^13^, which is consistent with the expected range for Akhiezer damping, and the dependence of *Q* on temperature and geometry is consistent with expectations for Akhiezer damping. These results thus provide the first clear, direct detection of Akhiezer dissipation in a MEMS resonator, which is widely considered to be the ultimate limit to *Q* in silicon MEMS devices.

## Introduction

Silicon MEMS are ubiquitous in consumer electronic devices and in automotive safety systems^1^. Microphones, pressure sensors, accelerometers, gyroscopes, and clocks are present in automobiles, smartphones, smart appliances, fitness and activity monitors, and many wearable electronics products, many billions of which are manufactured and sold every year^2^. A significant fraction of these devices relies on micromechanical structures operating in a vibrational mode, with their performance governed by the dynamical properties of the resonating element. For example, MEMS gyroscopes rely on resonant movement of a suspended mass in one axis, which couples through the Coriolis force to resonant movement in an orthogonal axis upon rotation. The resolution of MEMS Coriolis gyroscopes can be limited by thermomechanical noise arising from energy dissipation in the sense mode^3^, which scales as $\frac{1}{\sqrt{Q}}$. MEMS resonators are also used as time references, with short timescale errors limited by thermomechanical noise arising from energy dissipation^4^. Therefore, it is increasingly important to understand the fundamental limits to energy dissipation in these devices.

The quality factor, *Q*, of a resonator is a dimensionless parameter that indicates the rate at which energy stored in the resonant mode is lost. It is defined as the ratio of energy stored in the system to the energy dissipated per vibration cycle (Eq. 1); higher *Q* values therefore correspond to resonators with slower energy dissipation mechanisms:

1 $$Q=2\pi \,\frac{{\rm{Energy}}\,{\rm{stored}}\,{\rm{in}}\,{\rm{the}}\,{\rm{system}}}{{\rm{Energy}}\,{\rm{dissipated}}\,{\rm{per}}\,{\rm{cycle}}}$$

Several dissipation mechanisms can contribute to the loss of energy in MEMS resonators, contributing to the total *Q* as a reciprocal sum of terms^5^:

2 $$\frac{1}{{Q}_{total}}=\sum _{\frac{1}{{Q}_{factors}}}=\,\frac{1}{{Q}_{gas}}+\frac{1}{{Q}_{surface}}+\frac{1}{{Q}_{TED}}+\frac{1}{{Q}_{anchor}}+\frac{1}{{Q}_{Akhiezer}}+\frac{1}{{Q}_{other}}$$

In micromechanical resonators, the most important dissipation mechanisms have generally been identified as Gas damping, Thermo-Elastic Dissipation (TED), Anchor damping, and Akhiezer damping. For gyroscopes and timing devices, gas damping can be suppressed with vacuum packaging. TED is reduced by designing devices to minimize stress gradients within the resonating structure^6,7^. Anchor damping is normally suppressed by supporting the resonating elements at nodes of the vibrational mode and by using operating modes that are inertially symmetric. Surface losses have not been quantitatively modeled but are generally expected to scale with the surface-to-volume ratio; *Q* has been shown to increase with thickness for resonators in this limit, but no scaling with lateral dimensions is expected^5^. Akhiezer damping is expected to be important only for resonators operating at frequencies above 10 MHz with modes that are not expected to cause strong TED^8,9^. Researchers have identified MEMS resonators that approach this limit, but there has never been a direct, unambiguous demonstration of Akhiezer damping in a MEMS resonator^10,11^.

Importantly, however, many of these dissipation mechanisms have unique “signatures” in their temperature dependence that can be used to help identify the presence and magnitude of their contributions^12^, as discussed below.

## Q for Surface Losses

Surface losses have been discussed frequently in the literature and have been specifically identified as a dominant dissipation mechanism in some example devices^5,13^. In the early paper by Yasumura, a collection of silicon cantilevers with different thickness were characterized, showing *Q* which was lower than expected for TED and Anchor damping, and which was found to be proportional to thickness over a range of devices. This scaling was explained by assuming a constant area-proportional surface loss and a constant volume-proportional stored energy, such that *Q* is expected to be proportional to thickness for cantilevers with identical lateral dimensions and varying thickness. More recently, Villanueva reported on *Q* measurements in *SiN* membrane resonators, and identified surface losses as a dominant mechanism because of the linear scaling of *Q* with thickness, and independence of *Q* on lateral dimensions. These general scaling models for surface loss do not specifically identify the physical loss mechanism and therefore do not provide insight into the temperature dependence of such surface losses.

**Figure 1:**
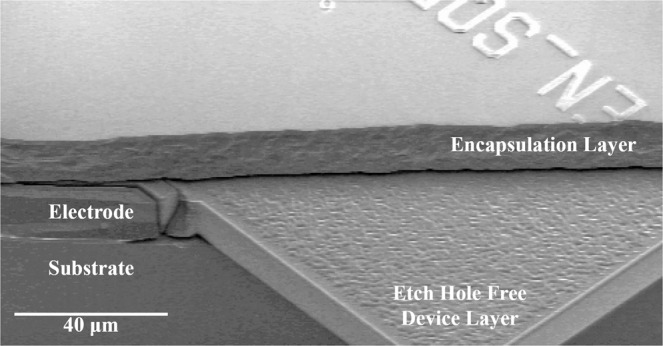
SEM image of an encapsulated Lamé-mode resonator after partial breakage of the die; this image shows the top encapsulation layer, the electrode and device layer, and the thick substrate underneath.

It is important to note that the encapsulation process used for our devices, described below, incorporates annealing at 1100 °*C* for as much as an hour after the seal is completed, with *H*_2_ residual gas trapped in the encapsulation. This treatment is known to cause a substantial smoothing of exposed *Si* surfaces, effectively eliminating roughness down to nm-scale on the surfaces of these devices as in Fig. 1. As such, we do not expect strong surface losses in the devices used in this study. The elimination of roughness in these devices should also suppress any additional “surface TED” that can arise from surface stresses localized near small-scale features or asperities on device surfaces^14^.

## Q(T) for Anchor Damping

Anchor damping is commonly understood to be determined by the forces and moments that a resonator’s oscillation exerts on its anchors; energy from the oscillation can be absorbed by the anchor itself or dissipated in the substrate as shear and normal stress waves^15–19^. Several early models for anchor damping predict that it will scale with the dimensions or aspect ratios of geometric parameters associated with the size of the resonator or anchor. For example, Hao^16^ has found that for a uniform rectangular beam of length *L* and width *w*, anchor damping scales as ${\frac{L}{w}}^{3}$, independent of the elastic modulus.

More recently, anchor damping models have been proposed that are based on the placement of a “perfectly matched layer” (PML)^20^, which describes a hypothetical boundary through which all strain energy that passes is lost. In either class of descriptions, the strength of anchor damping is treated as independent of temperature, save for the very weak temperature dependence of elastic constants or very small geometric changes from thermal expansion.

**Figure 2:**
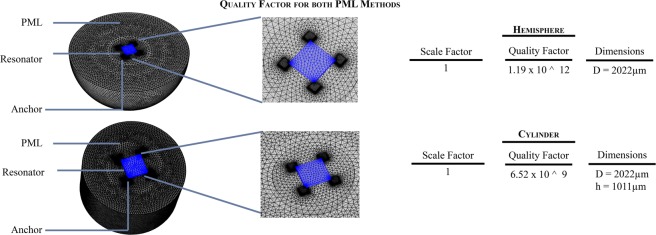
Anchor loss model in COMSOL Multiphysics, where the blue region represents the resonator and the grey region is the PML.

To estimate the possible role of anchor damping in these devices, we utilized a Finite Element Analysis (FEA) model for a PML available in the COMSOL Multiphysics Package. In this model, the PML was built using two different approaches, a hemispherical PML and a cylindrical PML. The hemispherical PML has a diameter *d* of 2022 μm, while the cylindrical PML has a 2022 μm diameter *d* and a 1011 μm height *h*; these dimensions were selected to be 20% greater than the wavelength in silicon at 10 MHz (843 μm)^17,20,21^. The PML model is presented in Fig. 2. The model predictions for quality factor associated with anchor damping in these devices ranged from 10^9^ to 10^12^. It must be noted that the PML simulation contains many free parameters, such as the “scaling factor,” the shape, and the location of the PML, that can be manipulated. In our experience, reasonable choices of these parameters consistently lead to results in the range given above.

## Q(T) for TED

In contrast to the loss mechanisms considered so far, TED is an intrinsic energy dissipation mechanism which is dependent on the material properties. TED is governed by coupling between temperature gradients and strain gradients through the thermal expansion coefficient. The governing equations of mechanical (wave equation) and thermal (heat conduction equation) behaviors in solids can be described by the following coupled equations^6,7,22,23^:

3 $$\rho \,\frac{{\partial }^{2}u}{\partial {t}^{2}}=E\,\frac{{\partial }^{2}u}{\partial {x}^{2}}+\frac{\alpha E}{(1-2\nu )}\frac{\partial T}{\partial x}$$

4 $$C\rho \,\frac{\partial T}{\partial t}=\kappa \frac{{\partial }^{2}T}{\partial {x}^{2}}-\frac{\alpha E{T}_{o}}{(1-2\nu )}\frac{\partial }{\partial x}\frac{\partial u}{\partial t}$$

where *u* is the displacement, *E* is the Young’s modulus, *ν* is the Poisson’s ratio, *c* is the heat capacity, *T*_*o*_ is the average temperature, *κ* is the thermal conductivity, *ρ* is the density, and *α* is the thermal expansion coefficient (CTE). In this system, the CTE determines the strength of the coupling between thermal and mechanical gradients; if CTE goes to zero, the coupling disappears and TED would be disabled as a loss mechanism.

A solid with a positive CTE under compressive strain will experience a temperature increase, while the same solid under tensile strain will undergo temperature decrease. Materials with a negative CTE will experience the opposite temperature change with applied strain, and materials with a CTE equal to zero experience no temperature change. Energy dissipation from TED arises from strain gradients that induce temperature gradients and thus irreversible heat flow. Consequently, TED should be a strong contributor to the overall energy dissipation in all resonators with vibrational modes that exhibit strong strain gradients, such as the bending modes of simple tuning forks and the stress concentrations exhibited in Lamé-mode resonators with arrays of etch release holes^24,25^.

In the simple case of a rectangular cantilever beam anchored at one end, the full analytical expression for TED originally derived by Zener^22^ is given by

5 $${Q}_{TED}=(\frac{{C}_{p}\rho }{E{\alpha }^{2}{T}_{o}})\,\frac{1+{(\omega \tau )}^{2}}{\omega \tau },\,\tau ={(\frac{b}{\pi })}^{2}\frac{{C}_{p}\rho }{\kappa }$$

where *C*_*p*_ is the specific heat capacity at constant pressure, *ω* is the angular frequency, *b* is the width of the beam in the direction of flexing, and *τ* is the time constant of the thermal mode. This specific analytical expression is only accurate for the simple cantilever beam, but the governing equations can be solved numerically for arbitrary resonator geometries, and the general observation that TED depends significantly on several materials properties, some of which have strong temperature dependences, and that TED is disabled whenever CTE = 0 arise from the general representation in Eq. 5. If the temperature dependence of the materials parameters is accurately known, numerical solutions can provide accurate predictions of the temperature dependence of TED in resonators^20,26^ for some specific resonator geometries.

**Figure 3:**
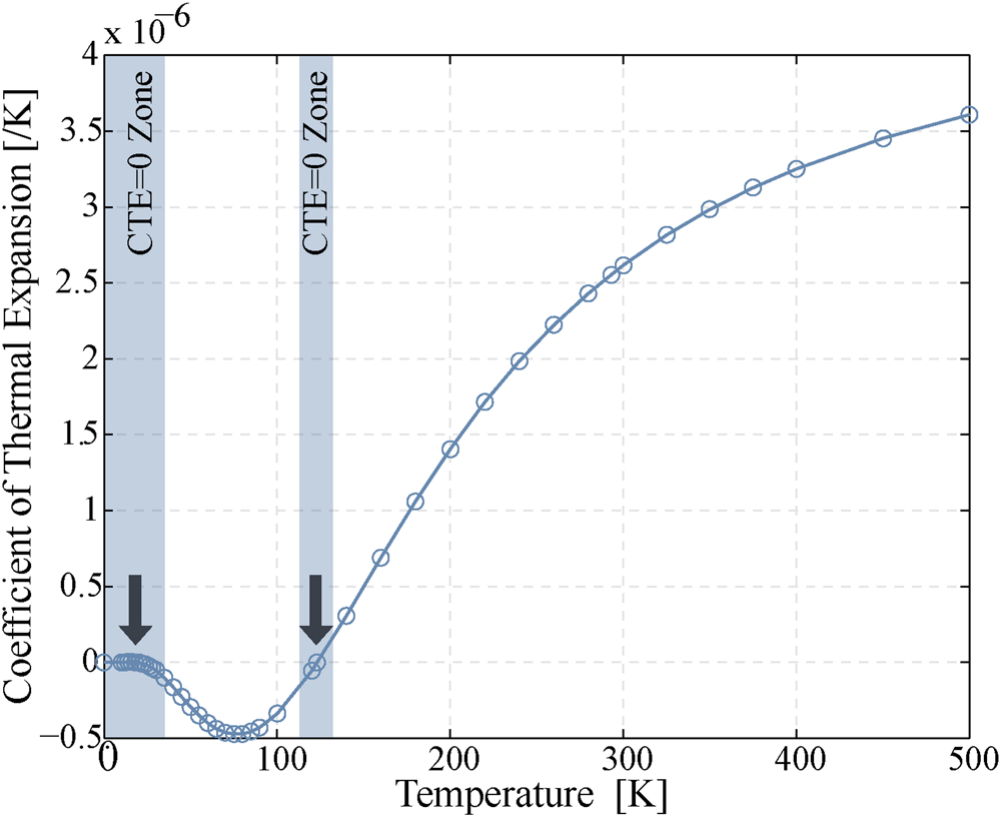
Plot of the temperature dependence of the coefficient of thermal expansion (CTE) for undoped silicon, from above room temperature to 0 K. At ~120 K, the CTE crosses zero, changing from positive to negative before returning to zero at the lowest temperatures.

As has been known for some time, the CTE of a material is itself a function of temperature. For undoped silicon, the temperature dependence of the CTE is complex, with the unusual feature that the CTE changes from positive to negative at approximately 120 K and asymptotically approaches zero below 20 K (Fig. 3)^27^. This zero-crossing in CTE(T) at 120 K is of interest for our research, because the contribution to energy loss from TED disappears as CTE(T) vanishes.

## Q for Phonon-Phonon Scattering

As described above, TED occurs due to spatial variations in stress, leading to temperature gradients and heat flow. In addition to TED, there are loss mechanisms associated with scattering of the acoustic phonons associated with the resonant mode with thermal phonons; these scattering processes can be divided into two regimes, the Landau-Rumer regime and the Akhiezer regime^28,29^. In the Landau-Rumer regime^30^, acoustic phonons scatter off the thermal population of phonons in individual bulk longitudinal modes, yielding a *Q* that is independent of the resonator frequency but strongly dependent on temperature (∝*T*^−4^)^31^. In the Akhiezer regime, the stresses induce distortion of the phonon energy spectrum away from the equilibrium Boltzmann distribution. This distortion of the energy distribution relaxes through scattering of acoustic phonons with the ensemble of thermal phonons in the material. The relaxation of the phonons from the perturbed state to the equilibrium state adds entropy and removes energy from phonon modes with a characteristic time constant known as the thermal phonon relaxation time, *τ*_*t*_^29^. This relaxation time determines in which regime a device is: if the acoustic wave period is shorter than the phonon relaxation time, it is in the Landau-Rumer regime; if the wave period is longer, then the system is in the Akhiezer regime. The MHz devices in this study are comfortably in the Akhiezer regime at all measured temperatures.

## Q(T) for Akhiezer Damping

Akhiezer damping, first identified by Akhiezer in 1965, arises from phonon scattering processes in silicon, independent of geometry^29^. Since Akhiezer’s discovery, the absorption of acoustic waves by Akhiezer damping has been the subject of extensive research. Bommel and Drasfeld [1960]^32^ developed an expression for attenuation of elastic waves that assumes the dominant heat flow takes place between two phonon branches; Woodruff and Ehrenreich [1961]^8^ modified this expression by solving the Boltzmann transport equation; and Mason and Bateman [1964]^33^ introduced a nonlinearity parameter *D* for the attenuation coefficient due to Akhiezer damping and reported early preliminary experimental results for silicon and germanium. Lambade (1995)^34^ introduced the integration schemes to include phonon scattering to arbitrary directions, and Iyer and Candler (2015)^35^ provided an analytical expression for the quality factor due to anharmonic phonon-phonon dissipation that induces the anisotropic energy storage and loss in a cubic semiconductor or dielectric crystal. The quality factor of an Akhiezer-limited resonator is given by:

6 $$f\cdot Q=\frac{3\rho {c}^{2}}{2\pi {\gamma }_{eff}^{2}{C}_{v}T{\tau }_{t}}=\frac{\rho {c}^{2}{c}_{D}^{2}}{2\pi {\gamma }_{eff}^{2}\kappa T}$$

where *c* is the velocity of the acoustic wave, *C*_*D*_ is the Debye velocity, *C*_*v*_ is the heat capacity at constant volume, *T* is the temperature, and *γ*_*eff*_ is the effective Grüneisen parameter. The Grüneisen parameter depends on the mode shape and the anisotropy of the material and is often left as a free parameter for fitting; we use as described for the Lamé-mode in Iyer and Candler 2015^35^. For silicon^9,28,31,35,36^, order of magnitude estimates of these parameters put the *f* · *Q* product at around 10^13^.

Several theoretical studies have focused on Akhiezer damping near room temperature; however, there remains significant difficulty in direct detection of Akhiezer damping because of other stronger dissipation mechanisms present in many practical devices, and interference from these other mechanisms has precluded experimental observation over a wide range of temperatures. Nevertheless, Akhiezer damping is widely regarded as imposing the upper limit to the achievable *f* · *Q* product of MEMS resonators^9,30,35^, and as such, there remains a compelling need for direct observation of Akhiezer damping in a device that is dominated by this effect. Such an opportunity would enable the first full experimental characterization and accurate understanding of the Akhiezer effect.

## Experimental Methods

**Figure 4:**
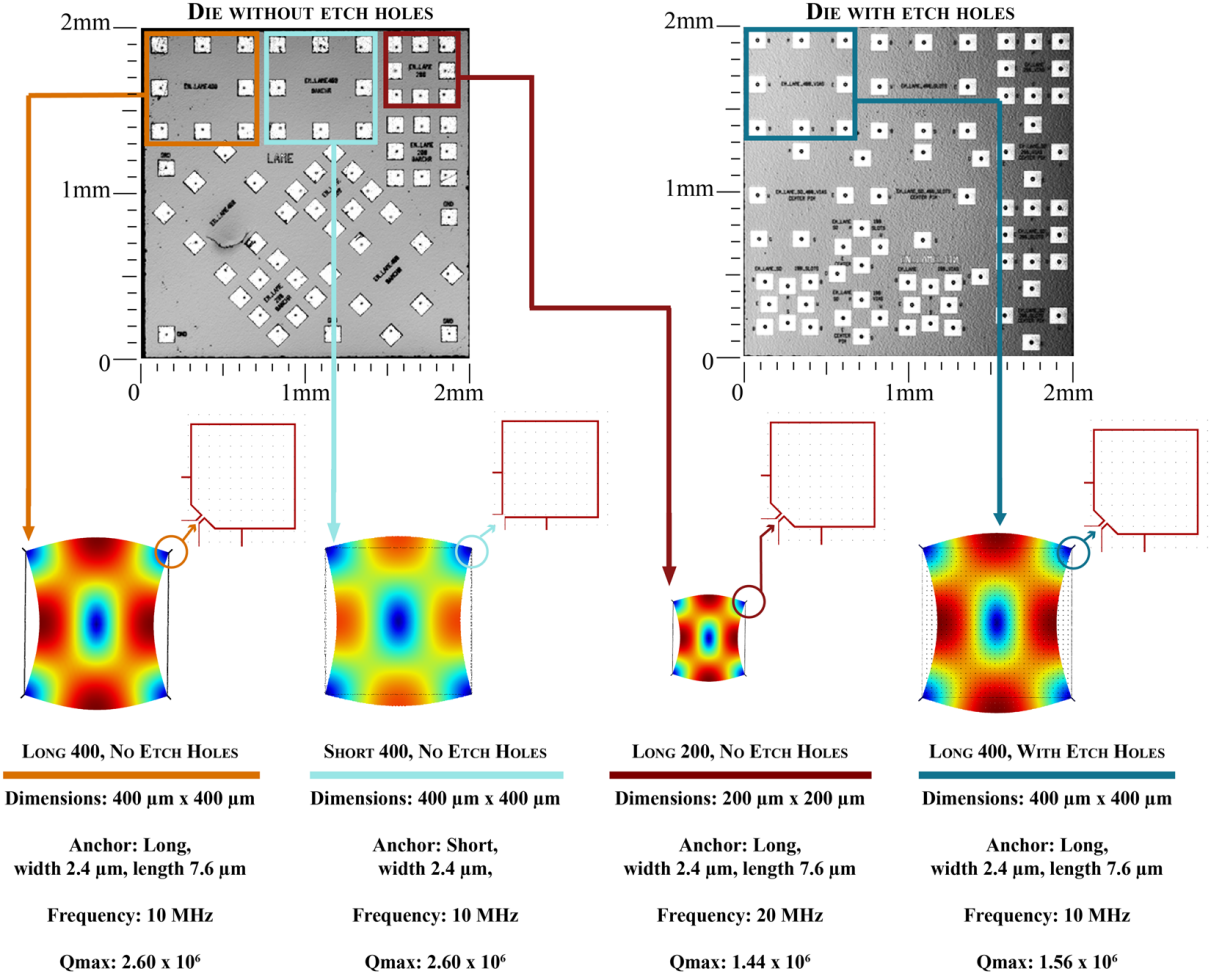
Details of the fabrication, mode shapes, anchors, and device’s specifications used in this study.

In this work, we studied a family of square Lamé-mode resonators with two different anchor designs (“short” and “long,” Fig. 4), two different sizes (400 μm × 400 μm and 200 μm × 200 μm), and that included resonators with and without etch release holes. These resonators are connected to the substrate through thin tethers located at the corners of the square plates, which are nodes of the Lamé–vibrational mode. This combination of designs allowed us to account for different dissipation mechanisms, such as TED, by identifying the *Q*(*T*) signatures present for each design. The geometric shape of the Lamé mode is shown in Fig. 4. At room temperature, these resonators have Lamé modes at 10 MHz for the 400 μm devices and 20 MHz for the 200 μm devices and *Q* values of 10^5^ for devices with etch holes, 2 × 10^6^ for the 400 μm devices without etch holes, and 10^6^ for the 200 μm devices without etch holes. The resonators without etch holes exhibit *f* · *Q* products of 2 × 10^13^ at room temperature, which is well within the range of highest–ever observed room temperature *f* · *Q* products.

To investigate the temperature dependence of dissipation in our MEMS resonators, we designed and built an experimental apparatus that allows testing of resonators while sweeping temperature from room temperature to less than 80 K and back^25^. The apparatus consists of a turbo-pumped vacuum chamber that allows mounting of a resonator die in a package that is clamped to a copper block through which liquid nitrogen is flowed. Electrical connections are made to the resonator die through a vacuum feedthrough. For all experiments in this study, *Q* values were determined by measuring the free ring-down responses, which simultaneously determines the frequency of the resonator as well as the decay time in the amplitude of oscillation. This approach allows accurate determination of the energy loss even if the frequency changes on the timescale of the experiment. Using this approach, we measured the temperature dependence of *Q* for all devices described in this study. Experimental details related to the operation of the resonators, initiation of the ring down measurement, and processing of the data have been discussed previously^37^.

The resonators used in this study were fabricated using our epitaxial polysilicon encapsulation (Epi-seal) process^38^, which has been shown to provide a hermetically-sealed environment for the resonator with a base pressure near 0.1 Pa, comprised of H_2_ gas. A variant of this process is used by SiTime to build MEMS-based timing elements for consumer electronics applications^39^, and the excellent repeatability and stability of devices fabricated in this process are critical to our work as well. The specific process used for these devices includes process variations that enable release of large-area resonating structures without release etch holes, enabling exploration of devices with and without them^38^. Resonators fabricated using the Epi-seal process exhibit long-term frequency stability of better than 30 ppb over 1 year, with corresponding stability in measurements of energy dissipation^38^.

Our devices operate in the Lamé mode, wherein gas damping has been shown to be negligible due to the very small displacements and high operating frequency of the bulk-mode^25,38^. In addition, we have experimentally confirmed that there is no dependence of the ringdown time constant on the bias voltage in these devices, which demonstrates that the electrical damping sometimes present in MEMS resonators, which can give rise to ohmic losses associated with motion-induced oscillating currents propagating through the devices^40^, is not an important contributor to our measured *Q*.

Based on these initial results, TED, anchor damping, and Akhiezer damping are expected to be the only important contributors to dissipation in these Lamé-mode devices. We also discuss the possible role of surface losses in these experiments below.

### Lamé-mode Resonators with Etch Holes

It has been shown that TED is the dominant source of energy loss in bulk mode resonators fabricated with etch holes. The introduction of etch holes in these resonators yields highly-localized concentrations of stress, and the resulting stress gradients dramatically strengthen TED^24,38,41^.

Beginning with the Lamé-mode device with etch holes, we measured *Q*(*T*) down to cryogenic temperatures. If, as we expect, TED is important in these devices, measurements to low temperatures should reveal several features:

- Starting from room temperature, the *Q* of TED-limited resonators should increase quickly as temperature is reduced^25^.
- As temperatures approach 120 K from above, we expect that the CTE approaches zero, and that the contribution from TED will disappear altogether. Below 120 K, CTE regains a finite value, so TED will reappear. The combination of these effects will produce a peak in the temperature dependence of the *Q* near 120 K.
- At the peak, TED can be entirely neglected as a dissipation source.

**Figure 5:**
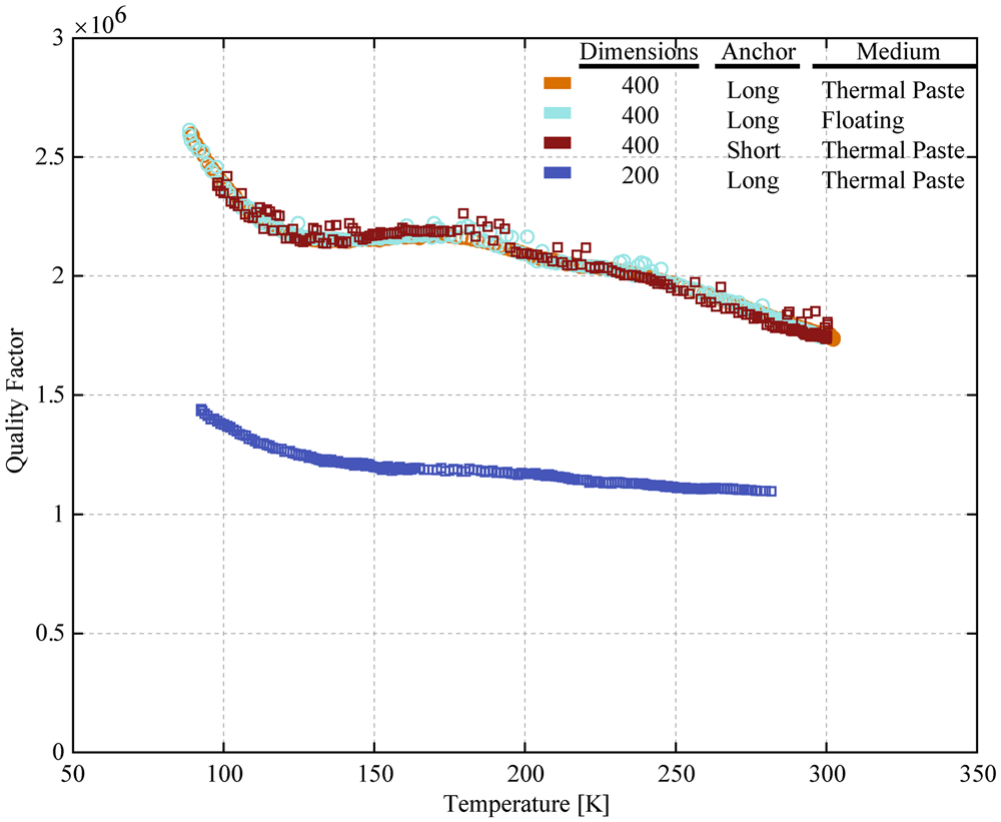
Measurements of *Q*(*T*) for 400 μm × 400 μm Lamé-mode devices with the long anchor configuration from room temperature to below 100 K. We see a peak in *Q*(*T*) at 120 K which is characteristic of a resonator dominated by TED, combined with some other dissipation mechanism that limits *Q* to 1.6 × 10^6^ at 120 K, where TED becomes negligible.

Figure 5 shows experimental *Q*(*T*) measurements for 400 μm × 400 μm Lamé-mode resonators with etch holes fabricated and operated as described above. These devices were mounted using two different mounting configurations. The first used a H20E epoxy silver paste from Epotek^42^ to attach the die to the package, and the second used a “floating die” method, which is implemented by suspending the die only by the wirebonds with no final adhesive^43^. In each of these measurements, we observed a room temperature *Q* of near 10^5^, rapidly increasing to a peak *Q* near 1.6 × 10^6^ at 120 K, and falling back down to less than 1.4 × 10^6^ at the lowest temperature measured. This *Q*(*T*) signature is consistent with what we expect for a device that is dominated by TED at most temperatures, but encounters some other upper limit to *Q* at 120 K when *Q*_*TED*_ diverges.

### Lamé-mode Resonators without Etch Holes

To understand other contributions to energy dissipation, we built and tested another set of Lamé-mode resonators with no etch release holes. By eliminating the etch holes, we expected to achieve a significant reduction in the strength of TED, allowing us to directly observe other dissipation mechanisms, such as anchor damping and Akhiezer damping. Prior work in our group^38^ and by others^24,44^ has shown that the *Q* measured at room temperature for devices without etch holes may be as much as 20X larger than for similar devices with etch holes.

**Figure 6:**
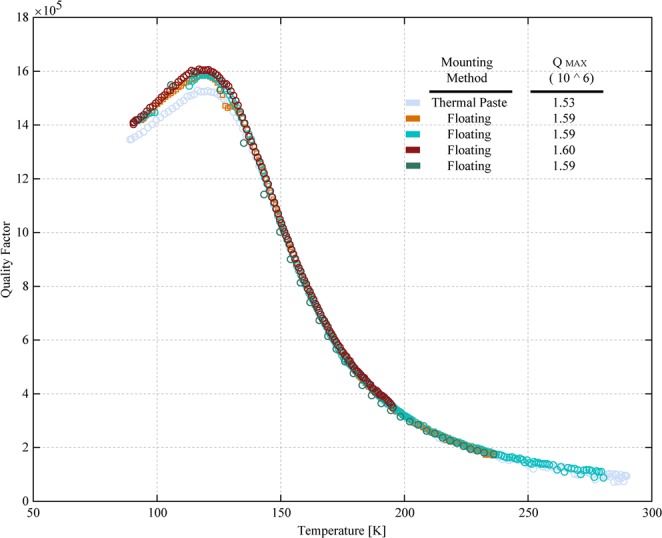
Measurements of temperature dependence of Q for Lamé-mode resonators without etch holes with different anchor geometries, different mounting approaches, and for two different sized devices.

An initial examination of these results (Fig. 6) leads to several observations. First, the absence of a peak in *Q*(*T*) near 120 K indicates that TED is not a significant source of dissipation in any of these devices. This result was also confirmed by TED simulations performed using COMSOL Multiphysics, where the predicted *Q* for TED was >10^11^. Second, the measurements of *Q*(*T*) do not appear to have any dependence on the anchor geometry or the die mounting method, so we believe that anchor loss is not a significant source of dissipation in these devices. Further, the observed *Q*(*T*) has a temperature dependence that is too strong to attribute the limiting loss mechanism to anchor damping. Finally, across the entire temperature range, *Q*(*T*) for the 400 μm resonator, which operates at 10 MHz, is roughly twice the *Q*(*T*) for the 200 μm resonator, which operates at 20 MHz. This observation is inconsistent with the expected scaling for surface losses, which would anticipate that *Q* would be the same for these devices with the same thickness and same surface area/volume ratio. Therefore, we conclude that surface losses are not a significant source of dissipation in any of these devices.

We do note that the peak *Q* for the device with etch release holes is 1.6 M, which is somewhat below the measured *Q* of 2.2 M for the device with the same dimensions but with no etch holes at the same temperature. One explanation for this difference is that the device with etch holes has significantly more surface area than the device with no etch holes, such that some very weak surface loss mechanism may be contributing. To cause the discrepancy, the additional surface loss would have to have a *Q* of about 6 M at 120 K, but would only be present in the device with etch holes.

**Figure 7:**
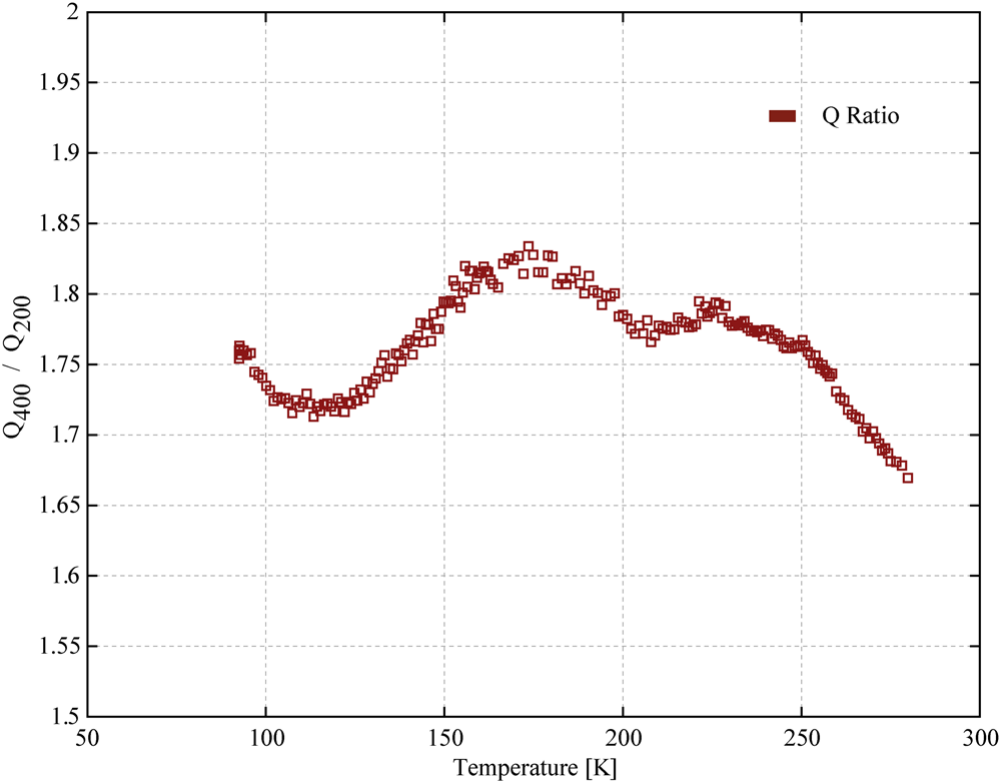
Ratio of Q(T) for the 200 μm and 400 μm Lamé resonators.

Based on these observations, we propose the dissipation in these resonators is dominated by Akhiezer damping. This assertion is based on the evidence that TED, anchor damping, and surface loss are not significant sources of dissipation, and that the measured *Q* at room temperature is in the range that previous work has asserted is consistent with approaching the “Akhiezer Limit”. Perhaps most importantly, we see that *Q* scales as 1/*f* for devices of different size which otherwise feature the same material, geometry, orientation, and anchors. Furthermore, *Q*(*T*) retains the expected 1/*f* scaling over a very wide range in temperature. Because *Q* depends on temperature only via the material properties, we expect that the temperature dependence is the same for both the 400 μm and 200 μm resonators. As a result, we interpret the near-2:1 ratio in *Q* for these two devices, which persists from 100 K to 300 K, as strong evidence of the first clear and direct detection of Akhiezer damping in a MEMS resonator. Figure 7 is a plot of the ratio of *Q*(*T*) for the 400 μm and 200 μm resonators as a function of temperature, showing that the Q(400)/Q(200) ratio is between 1.65 and 1.85 over this entire temperature range.

## Discussion

Having proposed that these experiments provide the first direct detection of Akhiezer damping, along with an observation of the temperature dependence of this dissipation mechanism, we would like to provide some physical explanation for the observed temperature dependence. Following Iyer and Candler^35^, we see that we must compute an effective mode-specific anharmonic Grüneisen parameter; in the case of a Lamé mode, *γ*_*eff*_ is given by $\langle {\gamma }_{i,5}^{2}\rangle$, where the brackets indicate an average over the various modes indexed by *i*, and 5 refers to the dispersion of each phonon mode with the shear strain, *ε*_5_. This parameter can be calculated from the modal Grüneisen parameters given in Mason and Bateman^33^; we obtain a value of 0.47, similar to the 0.51 calculated for *γ* at room temperature from the CTE. An average Grüneisen parameter, *γ*_*avg*_, can be estimated from the CTE by following Ishida (JPSJ 39, pg. 1282 (1975)) and Soma (JSPJ 42, pg. 1491 (1977)). This parameter is an average weighted by the corresponding modal heat capacities. The modal *γ*′*s* are taken to be temperature independent, with the heat capacities giving the overall temperature dependence. While *γ*_*avg*_ is positive at low temperature, where it is dominated by low-*Q* transverse acoustic phonons, it decreases with increasing temperature (as *γ* is negative for high-*Q* transverse acoustic phonons), eventually becoming negative. At higher temperatures, other phonon bands (all of which have *γ* positive) also come into play, leading to a *γ*_*avg*_ that becomes positive again and remains so. Using this *γ*_*avg*_ for the Akhiezer calculation would predict a divergence in *Q*_*Akhiezer*_ at the same temperature as observed for *Q*_*TED*_, which is inconsistent with our data. Using the *γ*_*eff*_ of Iyer and Candler corrects this discrepancy by accounting for the geometry of the resonant mode]. Unlike the *γ* calculated from CTE, however, this term is strictly positive, as it results from the sum of squared contributions from each mode. This is the underlying reason that *Q*_*TED*_ diverges when the CTE is 0, but *Q*_*Akhiezer*_ does not.

**Figure 8:**
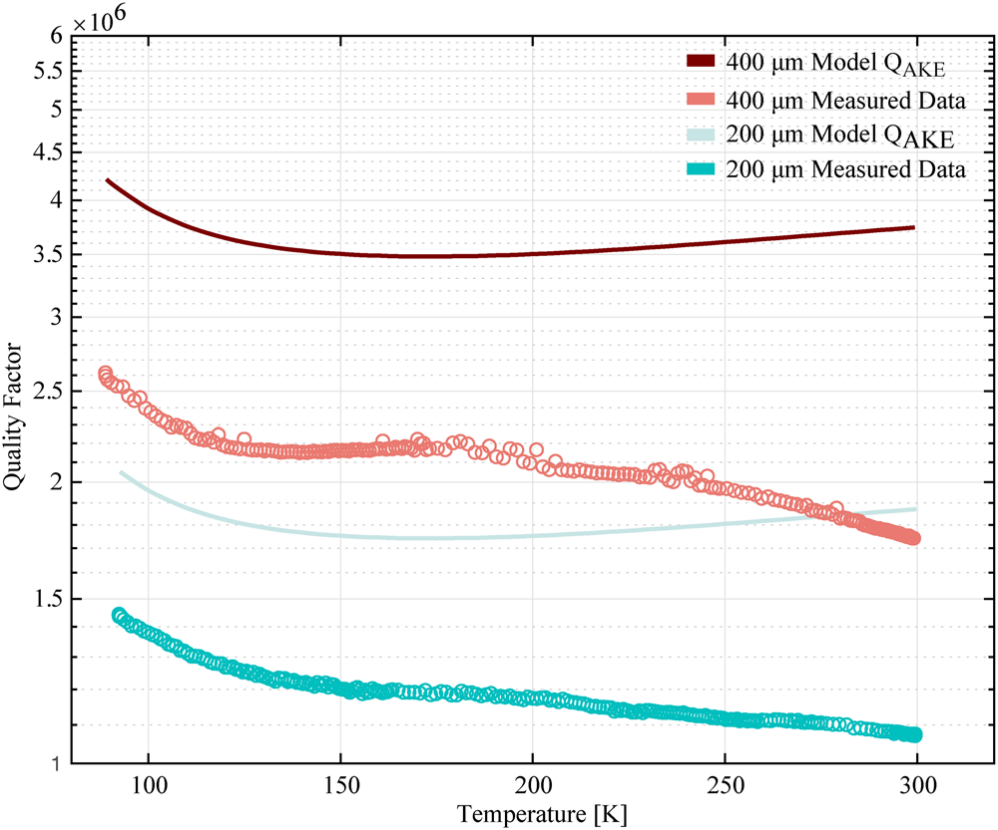
Measured and calculated *Q*_*Akhiezer*_ as a function of temperature for both 400 μm and 200 μm Lamé resonators.

We further assume that *γ*_*eff*_ is temperature independent, with the overall temperature dependence of the Akhiezer damping in our calculations determined entirely from the measured temperature dependence of the thermal conductivity^20,30^. We used published values for the density and acoustic wave velocity^45^. For the latter, we used the velocity of transverse acoustic waves in the 〈110〉 direction, which is the lowest acoustic wave velocity in bulk silicon; as such, this represents a lower limit on this model. Figure 8 shows a plot of our measured and calculated *Q*_*Akhiezer*_ as a function of temperature. We see that the model discussed above is roughly consistent with the experiments, and that perhaps some added source of dissipation, with a monotonically decreasing *Q*(*T*) is becoming dominant at *T* > 200 K for these resonators. As we see no evidence for TED in these devices, and we would expect anchor damping to be temperature-independent, this emerging dissipation mechanism is likely from some other source, as yet unidentified in these devices.

The Q(400)/Q(200) ratio shown in Fig. 7 is slightly below the 2.0 value that would be expected for devices that are completely dominated by Akhiezer damping. This raises the possibility that some other dissipation mechanism is contributing to losses in the 400 μm device, suppressing its measured Q by about 20%. A contribution from another mechanism with *Q* above 10 M across this temperature range could cause this reduction in the Q(400)/Q(200) ratio. Based on the experimental results available, we cannot definitively confirm the presence of another mechanism or make any specific determination that this is due to anchor dissipation or surface losses. Given the weak temperature dependence of the Q(400)/Q(200) ratio, any additional dissipation mechanism cannot have a strong temperature dependence, so anchor damping and surface losses are reasonable candidates.

Of course, it is clear that the trend in our approximate model is not a very good quantitative fit to the *Q*(*T*) data reported here. However, the overall qualitative agreement between the model and experiment in the magnitude of damping, over a wide range of temperatures, represents the first time that experimental data from a MEMS resonator has been suitable for such an analysis. Specifically, the measurements at different dimensions, with different anchors, and with/without etch release holes, carried out on several devices over a wide range of temperatures provides the most complete accounting for all of the dissipation mechanisms for a device operating near the expected *f* · *Q* limit.

## Conclusion

In this paper, we have shown measurements of the quality factor for Lamé-mode resonators over temperatures from room temperature down to below 100 K. These measurements enable clear identification of the mechanisms that contribute to energy loss. We confirm previous results that Lamé-mode resonators with etch release holes are dominated by thermoelastic dissipation, while Lamé-mode resonators without etch holes approach predicted limits from Akhiezer damping. By studying Lamé-mode resonators with different anchors and sizes, we have found evidence that these devices are, in fact, dominated by Akhiezer damping. We see the expected constant *f* · *Q* scaling among our devices and observe that this scaling is maintained across the entire temperature range measured and for devices of different *f*. Therefore, we offer that our measurements of *Q*(*T*) represent the first clear and direct observation of Akhiezer damping, including a measurement of the temperature dependence of *Q*_*Akhiezer*_ over this range of temperatures. In addition, we have modeled this temperature dependence with estimates of parameters including a mode–specific anharmonic Grüneisen parameter. Our results show the importance of measuring *Q*(*T*) and comparing it with temperature signatures for known dissipation mechanisms.
